# Pertussis in infants, in their mothers and other contacts in Casablanca, Morocco

**DOI:** 10.1186/s12879-019-4680-1

**Published:** 2020-01-14

**Authors:** Khalid Katfy, Idrissa Diawara, Fakhredine Maaloum, Siham Aziz, Nicole Guiso, Hassan Fellah, Bouchra Slaoui, Khalid Zerouali, Houria Belabbes, Naima Elmdaghri

**Affiliations:** 1 Department of Microbiology, Faculty of Medicine and Pharmacy, 19 rue Tarik Bnou Zyad, 20360 Casablanca, Morocco; 2 Bacteriology-Virology and Hospital Hygiene Laboratory, University Hospital Centre Ibn Rochd, 1, Rue des Hôpitaux, 20100 Casablanca, Morocco; 3 Department of Immunology, Faculty of Medicine and Pharmacy, 19 rue Tarik Bnou Zyad, 20360 Casablanca, Morocco; 4 Abderrahim Harouchi Pediatric Hospital, rue Mohamed El Faidouzi, -ex Jenner Quartier: Hôpitaux -, Casablanca, Morocco; 5 Molecular Prevention and Therapy of Human Diseases, Institut Pasteur, 25 rue du Dr Roux, 75015 Paris, France; 6 Faculté des Sciences et Techniques de Santé, Mohammed VI University of Health Sciences (UM6SS), Casablanca, Morocco

**Keywords:** Pertussis, *B. holmesii*, *B. Parapertussis*, Household contacts, Anti-PT IgG/IgA

## Abstract

**Background:**

In recent decades, there has been a marked increase in the number of reported cases of pertussis around the world, and pertussis continues to be a frequently occurring disease despite an effective childhood vaccination. This study aims to determine the role of household contacts of children diagnosed with pertussis in Casablanca Morocco.

**Methods:**

From November 2015 to October 2017, children suspected of whooping cough that consulted Ibn Rochd University hospital at Casablanca with their household contacts were enrolled in the study. Nasopharyngeal (NP) samples of the suspected children were analyzed by culture and RT-PCR. For the household contacts, NP and blood samples were collected and analyzed by RT-PCR and specific detection of pertussis toxin antibodies by ELISA, respectively.

**Results:**

During the study period, the survey was carried out on 128 infants hospitalized for pertussis suspicion and their families (*N* = 140). *B. pertussis* DNA was specifically detected in 73 (57%) samples, coexistence of *B. pertussis* and *B. parapertussis* DNA in 3 (2.3%) samples, coexistence of *B. pertussis* and *B. holmesii* DNA in 10 (7.81%) and only one (0.78%) sample was IS *481* RT-PCR positive without the possibility of determining the *Bordetella* species with the diagnostic tools used. Confirmations of Pertussis infection in household contacts by culture, RT- PCR and serology were 10, 46 and 39%, respectively.

*B. pertussis* DNA was confirmed in the infants as well in their mothers in 38% of the cases. Co detection of *B. pertussis* and *B. parapertussis* DNA in 2% and co-detection of *B. pertussis* and *B. holmesii* DNA in 4%. *B. holmesii* DNA alone was detected in 5 NP samples of index cases and their mothers.

**Conclusions:**

The results of this study confirm that *B. pertussis* is still circulating in children and adults, and were likely a source of pertussis contamination in infants still not vaccinated. The use of RT-PCR specific for *B. pertussis* in the diagnosis of adults is less sensitive and should be associated with serologic tests to improve diagnosis of pertussis and contributes to preventing transmission of the disease in infants.

## Background

In recent decades, there has been a marked increase in the number of reported cases of pertussis around the world, and pertussis continues to be a frequently occurring disease despite an effective childhood vaccination [1]. Resurgence has been reported in many countries worldwide, even in countries with high vaccine coverage [2], probably due to multifactorial causes: atypical or non- characteristic symptomatology of classical pertussis in adolescents and adults, increased clinician awareness and reporting, contribution of sensitive biological diagnostic tools such as a sensitive and an easier real-time PCR (RT-PCR), low vaccine coverage, particularly for boosters [3, 4]. Studies of children’s family with confirmed pertussis disease has attracted global concern, it is useful to detect new cases independently of the symptoms they have. Indeed, pertussis in adults has been reported elsewhere in several studies [5]. The prevalence of pertussis in this age group remains underestimated [6], due to the variety of clinical symptoms, the absence or differences in diagnostic methods and case definitions used. Since the introduction of vaccination in young children, a change of transmission has been observed. Adults are now shown to transmit pertussis to their unvaccinated or partly immunized infants and children [7] [8];.

Epidemiological surveillance of pertussis in household requires the adaptation of highly relevant biological diagnostic tools, in particular specific PCR able to distinguish between *Bordetella* species [9] [10];. Currently available *B. pertussis* diagnostic methods include direct diagnosis such as culture, specific RT-PCR and indirect diagnosis such as the detection of anti-pertussis toxin antibodies [4]. As previously reported by several authors [11], culture and RT-PCR are specific and sensitive in infants and young children but less specific in adolescents and adults due to the carriage of *B. holmesii,* and less sensitive since often adolescents and adults are coming after more than 3 weeks of cough or already treated by macrolides [12] [13];. After three weeks of cases, pertussis can be diagnosed by quantification of anti-pertussis toxin antibodies by Enzyme linked immuosorbent assay (ELISA) [4]. Serologic diagnosis in infants is very rarely useful, due to the delay in antibodies levels elevation; moreover the infant’s serum may contain antibodies transmitted by the mother up to 6 months after birth [14].

In Morocco, Pertussis vaccination [pertussis whole cell vaccine (wP) in combination with diphtheria and tetanus toxoids (DTwP)] was introduced by the national immunization program (NIP) in the early 1980’s to prevent pertussis [15]. The Moroccan vaccination strategy includes a primary vaccination at 2, 3 and 4 months of age and two boosters at 18 months of age and 5 years of age [16]. The vaccine pertussis vaccine coverage exceeds 95% at the age of 24 months [17].

In Morocco, there are few epidemiological data on pertussis infection in household contacts and their involvement of pertussis diseases among children [16]. The purpose of this study was to determine the role of household contacts of children diagnosed with pertussis in Casablanca Morocco.

## Methods

### Study design

This cross-sectional study of whooping cough was conducted from November 2015 to October 2017 with the participation a public hospital network in Grand-Casablanca - Morocco, including all patients under 14 years and child’s household contacts. For each subject, the basic demographic and epidemiological data collected and recorded, such as sex, age, date of sampling, address, medical history associated with chronic diseases, and vaccination status.

The study was performed at the Microbiology Laboratory of Ibn Rochd University Hospital Centre of Casablanca (IR-UHC). In the IR-UHC, pediatric patients are managed at the Abderrahim Harrouchi Children University Hospital. All cases of serious diseases such as pertussis and complicated diseases in other hospitals are systematically transferred to IR-UHC.

### Laboratory methods

#### Samples

**-** Nasopharyngeal aspiration (NPA): All index cases and household contacts were sampled according to our previously published procedures [16].

**-** Blood sampling: Blood samples were collected in vacutainer and tested in all household contacts. The serum was separated directly or after blood sampling (24 h at room temperature). In our study, serum with hemolysis or volume less than 100 μL were not used for the study. All serum were stored at − 80 °C prior to analysis.

NP samples or blood samples taken from hospitalized patients and household contacts were sent to the microbiology laboratory of Ibn Rochd University Hospital in Casablanca at room temperature, accompanied by a fact sheet with all clinical and socio-demographic indications.

Mothers were automatically sampled with their children. Other members of the family have benefited of NP/serum samples only when the index case is positive and these household contacts have compatible signs such as prolonged cough.

Duplicates of the same person are excluded from the analysis.

#### Direct diagnosis

a) As for the direct diagnosis, we used our previously methods [16] for the bacterial culture of *Bordetella species* and real-time PCR for the detection of the presence of *Bordetella* strains harboring IS*481* (*Bordetella spp*), IS*1001* (*B. parapertussis*), *ptxA*-*Pr (B. pertussis)* and *h-IS1001* (*B. holmesii*).

b) *Indirect diagnosis methods*: Serology by ELISA (Enzyme Linked Immunosorbent assays).

In this study, measurement of antibodies to *B. pertussis* antigens was done by ELISA). Serum samples were analyzed by using a commercial kit (SeroPertussis™ Toxin IgA Kit and SeroPertussis™ Toxin IgG Kit –Savyon - Diagnostics Ltd) used for quantitative detection of IgG and IgA antibodies to Pertussis Toxin in human serum, and expressed in International Units per milliliter (IU/ml). The microplates were read on the ‘BIO-RAD PR2100 Microplate Reader’ (manufacturer) instrument, designed to measure the optical density (OD) of fluid samples in 96 well microplates [18].

**Fig. 1:**
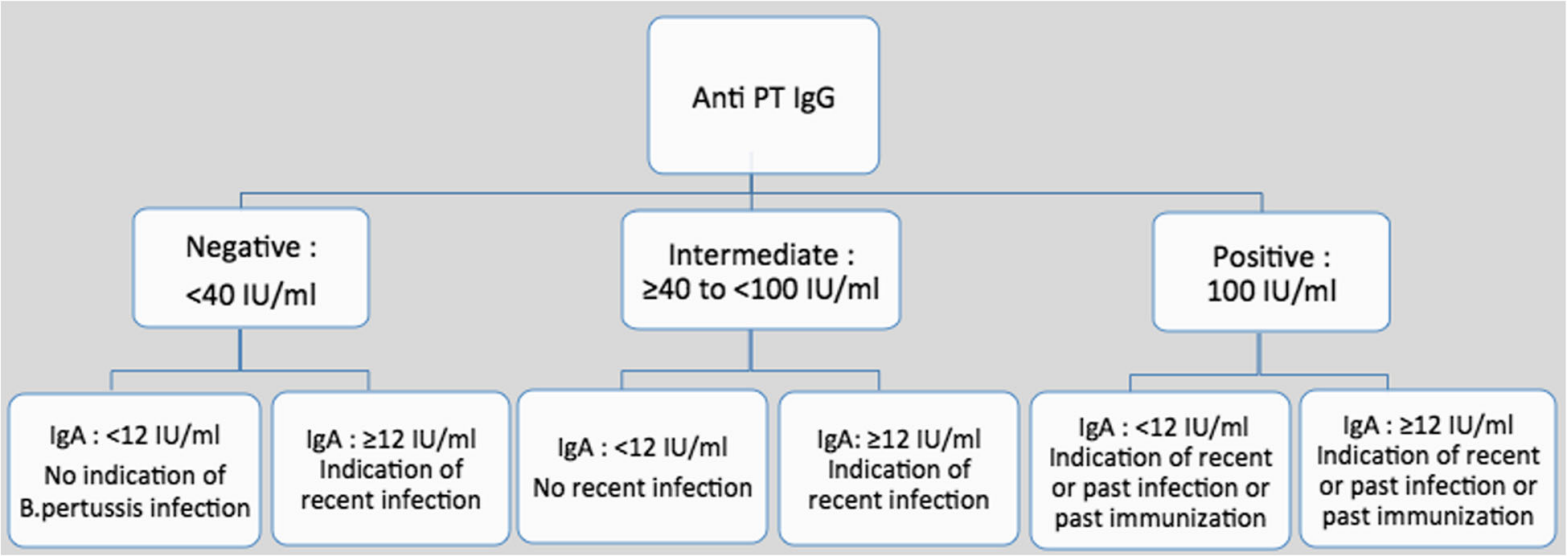
Interpretation of the results according to the IgG/IgA antibody profile in patient serum [19]

The interpretation of IgG-anti-PT antibodies was as recommended by manufacturer (Fig. 1). The IgG-anti-PT values below to 40 IU/ml were not indicator of recent contact [19] [20]. Whereas levels above > 100 IU/ml can be used as an indicator of recent contact with the bacteria. If diagnosis cannot be established with certainty (Single serum, Intermediate range), Savyon Diagnostics recommends testing IgA levels, which may serve as an additional test for equivocal results (> 40 and < 100 IU/ml).

### Data management and statistics

Data entry was performed using WHONET 5.6 software. Analyzes were performed using Epi info (CDC, Atlanta, Georgia), Microsoft Excel and the JASP software [21], a cross platform statistical software program with a state-of-the-art graphical user interface.

The statistical significance adopted for the study was 5% (*p-values* < 0.05).

## Results

During the study period, between November 2015 and October 2017, 268 NP samples were collected from infants consulted Ibn Rochd University hospital at Casablanca with clinical suspicion of pertussis disease and some of their household contacts.

**Fig. 2:**
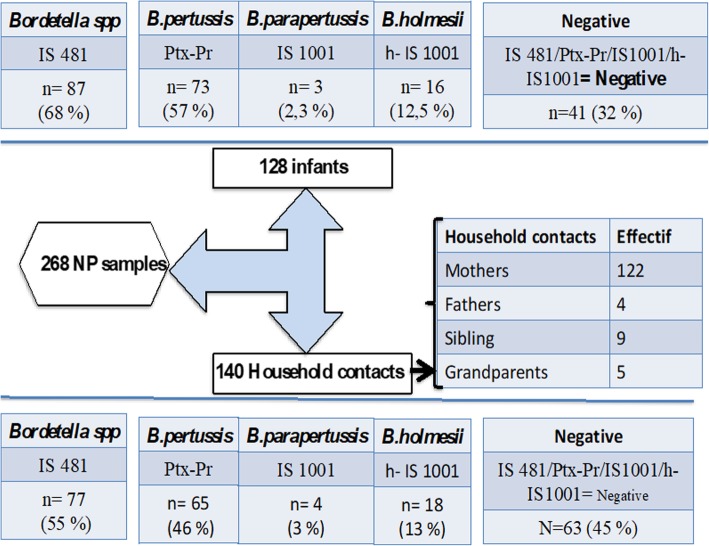
Results of confirming the diagnosis of pertussis in infants and household contacts

Hospitalized children were below 5 years of age, with an average of 60 ± 10 days and 87% (111/128) were under 2 months of age. Household contacts were essentially mothers (87%). The participation of other family members was very low with only 4 (3%) fathers, 9 (6%) brothers and siblings (five sisters, four brothers), and 5 (4%) grandparents (Fig. 2). Eighty-two percent of the NP samples included in this study were from infants admitted to Abderrahim Harrouchi pediatric hospital. The other Casablanca hospitals were poorly represented.

Clinically, 85.6% of patients diagnosed for pertussis in this study had common symptoms of typical pertussis, 14.3% of pneumo-pertussis, sometimes complicated with superinfection in 12% of cases, syncopal apnea in 6%, and cyanosis in 36%. Administration of antibiotics, mainly macrolide family, was noted in 82 from 87 (95%) infants diagnosed (Table 1). More than 65% of household contacts had no symptoms of whooping cough, 20% more than three weeks and 15% less than three weeks. Of these, 67% mothers had no symptoms of pertussis but were in contact with at least one person who was coughing at home.

**Table 1:** Clinical information of patients and their household contacts

	Infants diagnosed		Household contacts			
	Not confirmed (*n* = 41)	Confirmed (*n* = 87)	Mothers (*n* = 122)	Fathers (*n* = 4)	Siblings (*n* = 9)	Grandparents (*n* = 5)
Symptoms						
Coughingparoxysm	18 (44%)	40 (46%)	10 (8%)	0 (0%)	2 (22%)	1 (20%)
Coughing	21 (51%)	60 (69%)	45 (37%)	4 (100%)	6 (67%)	4 (80%)
Singing Rooster	27 (66%)	74 (85%)	1 (0,8%)	0 (0%)	1 (11%)	0 (0%)
Vomiting	23 (56%)	9 (10%)	9 (7%)	0 (0%)	3 (33%)	2 (40%)
Cyanosis	20 (49%)	30 (34%)	0 (0%)	0 (0%)	0 (0%)	0 (0%)
Apnea	4 (10%)	5 (6%)	0 (0%)	0 (0%)	0 (0%)	0 (0%)
Respiratory distress	23 (56%)	26 (30%)	37 (30%)	4 (100%)	7 (78%)	4 (80%)
Antibiotictreatment						
Yes	37 (89,7%)	82 (94,7%)	8 (6,9%)	4 (100%)	2 (22%)	2 (40%)
No	4 (10,2%)	05 (5,2%)	114 (93%)	0 (0%)	2 (22%)	1 (20%)
Hospitalization	37 (91%)	87 (100%)	0 (0%)	0 (0%)	1 (11%)	0 (0%)

Cultures of NP samples were performed from 51 of the 128 index cases and from 51 of the 140 contacts, other NP samples were not tested by culture for various reasons e.g., unavailability of culture media, samples improperly transported or stored. The culture was positive in 16% (8/51) of the index cases and 10% (5/51) of the contacts.

All samples were tested by RT-PCR, Among 128 NPA of infants included, IS *481* RT-PCR was positive in 68% (87/128), the majority was observed in unvaccinated children less than 2 months of age 74% (64/87). Other incompletely vaccinated 14% (*1*2/87) with one or two doses were aged between 3 and 14 months. *B. pertussis* DNA was specifically detected in 73 (57%) samples, coexistence of *B. pertussis* and *B. parapertussis* DNA in 3 (2.3%) samples, coexistence of *B. pertussis* and *B. holmesii* DNA in 10 (7.81%) and only one (0.78%) sample was IS *481* RT-PCR positive without the possibility of determining the *Bordetella* species with the diagnostic tools used. Six NPA were positive only for *B. holmesii* DNA only. *Bordetella* DNA was not detected in 41 (32%) samples. No co-infection between *B. parapertussis* and *B. holmesii* was found (Fig. 2).

**Fig. 3:**
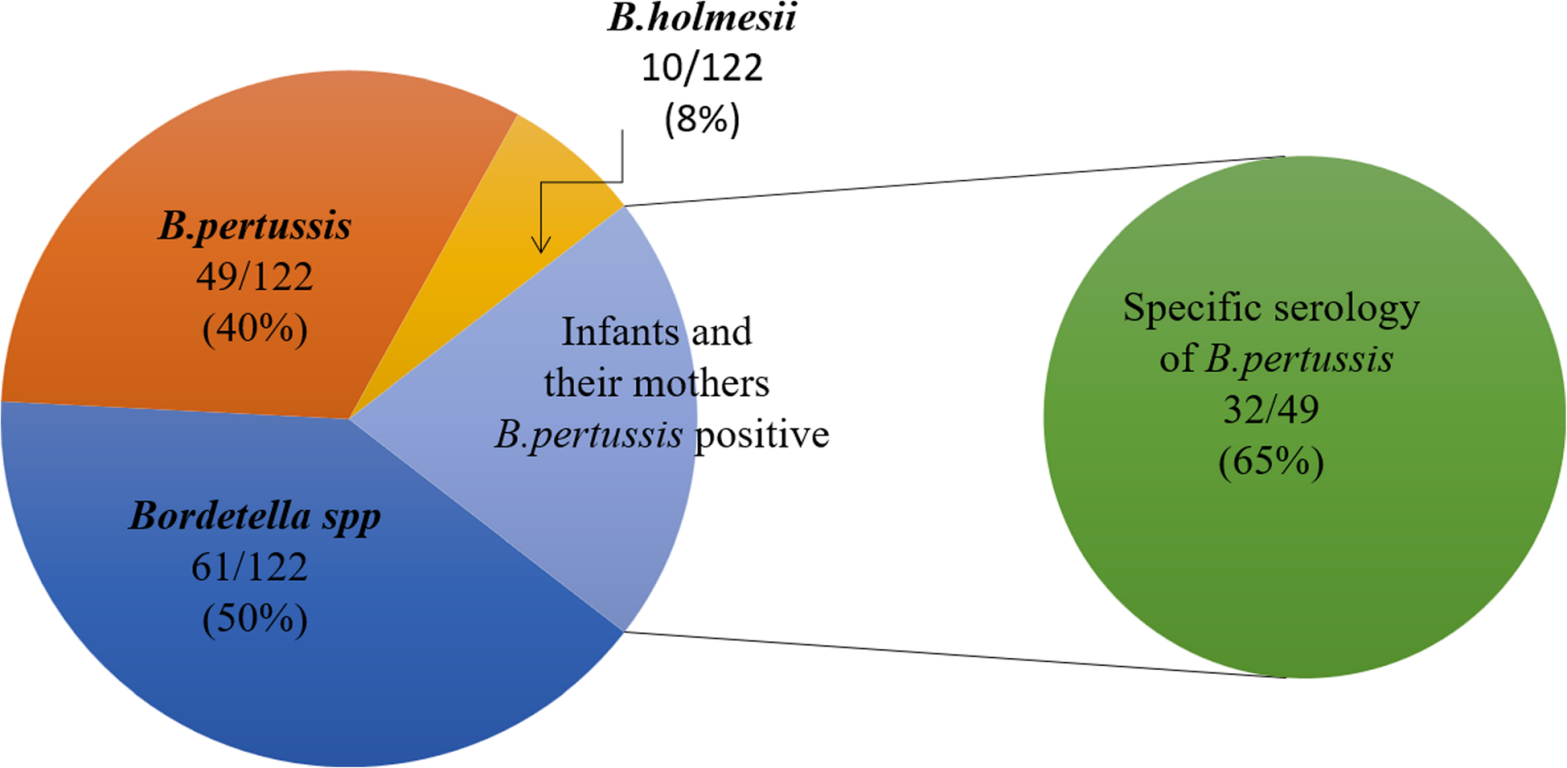
Results of pertussis in infants and their mothers

**Fig. 4:**
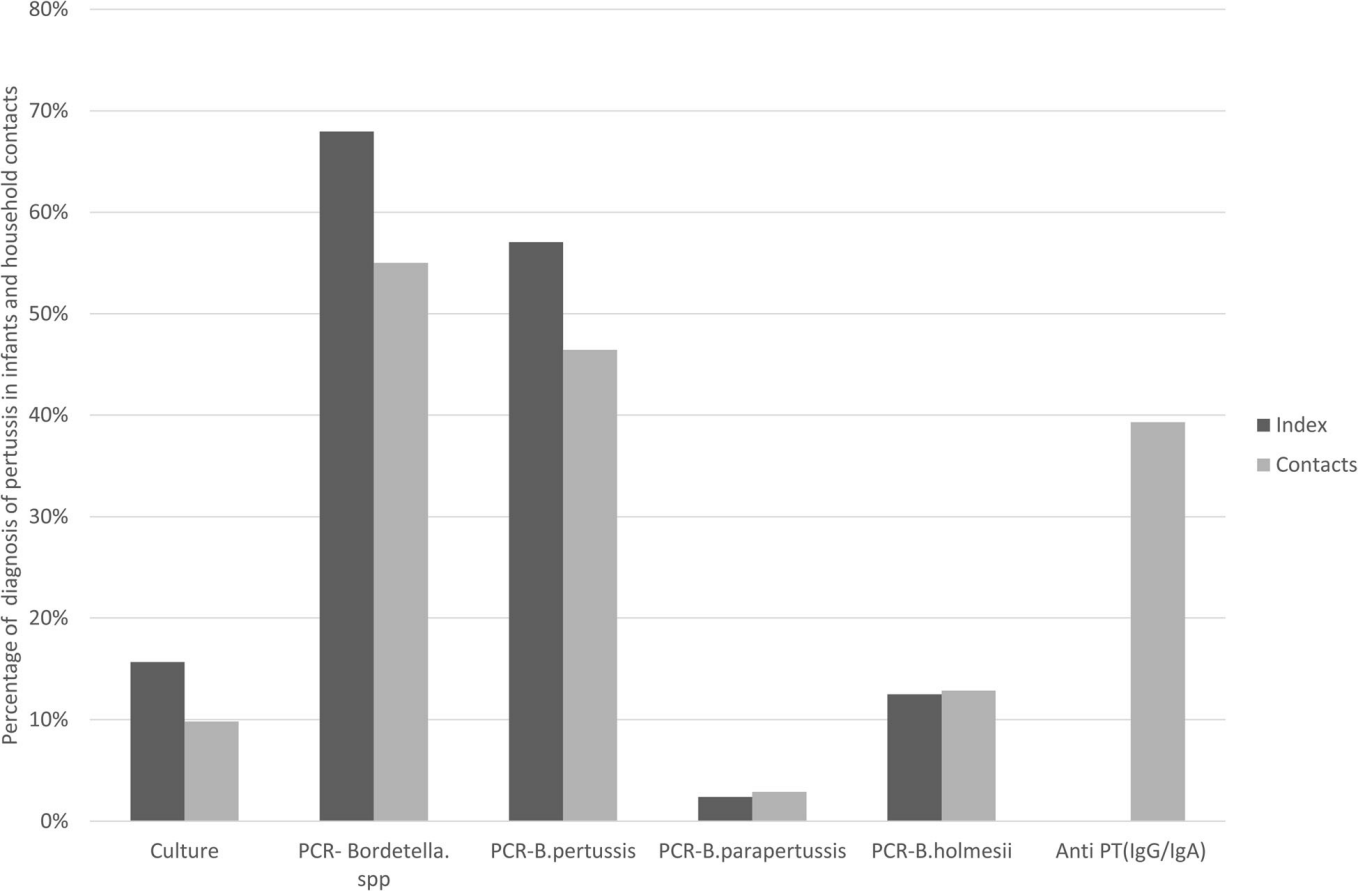
Results of pertussis in infants and their mothers according the different methods

No *Bordetella* DNA was detected in the NPA of the household members of the non-infected infants. Among the 140 NPs of household contacts, IS *481* RT-PCR was detected in 55% (77/140). *B. pertussis* DNA in 46% (65/140) samples, *B. parapertussis* in 3% (4/140) samples, *B. holmesii* in 13% (18/140) samples, and three NPs were positive by RT-PCR IS *481* alone and identified as *Bordetella spp*. The coexistence of *B. pertussis* and *B. parapertussis* DNA was detected in 3 (2%) samples, *B. pertussis* and *B. holmesii* in 6 (4%) samples. No co-infection between *B. parapertussis* and *B. holmesii* DNA was found. *B. holmesii* DNA was detected in 6 index cases and 5 of their mothers. Pertussis was confirmed by RT-PCR IS *481* in 64 from 122 (52%) mothers. Also other family members participating in this survey were not spared from the infection: 3 of 5 grandparents, 8 of 9 siblings and one of 4 fathers were also pertussis positive. Pertussis was confirmed in children and their mothers together by PCR for *Bordetella spp* in 50% (61/122), *B. pertussis* in 40% (49/122), and *B. holmesii* in 8% (10/122). No *B. parapertussis* was detected (Fig. 2). Of these, only 32 from 49 mothers were confirmed serologically (Figs. 3 and 4).

A total of 140 blood serum were analyzed only in household contacts with single serum because of parents refusal to return for a second blood test or technical difficulties in getting a venous blood sample. Serology confirmed infections in 55 from 140 (39%) household contacts by anti-PT (IgG and IgA) antibodies tests. Over 100 IU/ml of anti-PT IgG were measured in 5 serums and interpreted as an indicator of recent contact with the bacterium. Between 40 and 100 UI/ml in 17 serums, to analyze this intermediate results depend of Anti-pertussis IgA antibodies tests, 12 of them were considered as a recent *B. pertussis* infection. Anti-PT IgG > 40 and IgA > 12 in 15 cases, and anti-PT IgA > 12 UI/ml in 53 serums (Table 2).

**Table 2:** Results of interpretation of the serological profiles

IgG and IgA interpretation				
IgG results	IgA results	Indication of a recent *B.pertussis* infection, or recently vaccinated	Absence of recent infection, or past infection, or past immunization	Recent contact with the bacterium
Intermediate	Positive	12	0	0
	Negative	0	0	5
Highly Positive	Positive	3	0	0
	Negative	2	0	0
Negative	Positive	38	0	0
	Negative	0	80	0

Comparison of the serology and other diagnosis tools showed that all culture-positive were also positive by serology, and showed significantly higher levels of IgG (> 100 IU / ml) compared to negative cultures samples.

*B. pertussis* infection was confirmed by real time PCR and detection of anti-PT antibodies in 41 (29%) cases and only by RT-PCR in 22 (16%) of cases. In 12 (9%) cases, *B. pertussis* infection was confirmed only by detection of anti-PT antibodies in the serum of the patients. *B. holmesii* was found in 16 index cases and in 10 of their mothers, 4 of them were co-infected with *B. pertussis*, these mothers were serology negative (Table 3).

**Table 3:** Contribution of RT-PCR/ anti-PT antibodies tests for diagnosis pertussis in household contacts

IgG & IgA anti-PT	RT-PCR Ptx-Pr Positive in household contacts		
	Negative	Positive	Total
Indication of recent infection	12	43	55
No indication of *B. pertussis* infection	62	18	80
Recent contact with *B. pertussis*	1	4	5
Total	75	65	140

## Discussion

This study completes a previous study [16]. In the present study, we analyzed the household contacts with or without symptoms of whooping cough to determine their involvement in infant’s pertussis infection. The case definition of pertussis in adolescents and adults is based solely on clinical diagnosis in Morocco. However, experts underline that current clinical case definitions cannot universally be applicable, and that different age groups should be evaluated by different clinical criteria [22] [23]. Laboratory confirmation of *B. pertussis* infection is not routinely used; therefore the rate of *B. pertussis*/*B. parapertussis* infections is probably underestimated. Direct and indirect laboratory methods used in this study for pertussis diagnosis are available. Direct tests are (RT-PCR) and culture, whereas indirect tests measure specific anti-PT antibodies. The choice of the biological test depends on the age and the duration of symptoms: in neonates and young infants post-onset of symptoms, PCR and/or culture should be performed. Measuring antibodies to *B. pertussis* antigens is mainly meaningful on household members (vaccinated older children, adolescents and adults). In cases with less than two weeks of coughing, culture and PCR from nasopharyngeal samples should be done. If coughing lasted at least 2 to 3 weeks, the measurement of IgG-anti-PT should be sufficient [20].

During the study period, every infant diagnosed was mostly accompanied by his/her mother, and very few other household members very few people were able to participate. In this study, 51 infants and their 51 contacts benefited from culture. Pertussis was faintly confirmed in 8 infants and 5 mothers. Several studies have demonstrated low culture sensitivity to confirm a case of pertussis as compared to RT-PCR and blood tests [7]. The sensitivity of cultures in pertussis diagnosis depends on the duration of symptoms, age, antibiotics treatment and the vaccination status of the patient [24]. Despite the difficulties, culture remains the “gold standard” pertussis diagnosis method due to its high specificity, and is important for following the evolution of the bacteria, and for monitoring antibiotic sensitivity especially to macrolides [25].

The performance of PCR based exclusively on *IS*481 is highly sensitive and confirmed the circulation of *Bordetella spp* in 87 (68%) infants and 77 (55%) in their household contacts respectively. It was also reported that other *Bordetella species* sometimes associated with respiratory disease in humans, including *B. bronchiseptica* and *B. holmesii* harboring the IS481 sequence, which may lead to misidentification as *B. pertussis* [26] [27]. However, to differentiate *Bordetella species* and to avoid false positive results, we used other targets specific for *B. pertussis*, *B. parapertussis* and *B. holmesii* [28]. Our results showed the predominance of *B. pertussis* among NP samples from 73 (57%) infants and 65 (46%) of household members, it should be noted that 87% (76 of 87) were infants less than two months of age and didn’t receive any dose of vaccines according to the Moroccan vaccine strategy recommendation, leaving these children in danger of direct contamination. *B. parapertussis* was detected in 3 infants samples as well in the household contacts. In these infants samples, *B. pertussis* was also detected. *B. parapertussis* remains low compared to neighboring countries [29].

*B. holmesii* was detected in 16 NP samples from index cases and 18 of their contacts. Coexistence of *B. holmesii* and *B. pertussis* was observed in 10 cases with *B. pertussis*, as observed in other studies [9, 10, 16, 18].. In 6 index cases and 8 household contacts, unfortunately no other microorganisms were searched. For this reason we couldn’t determine whether *B. holmesii* was responsible of the cough or not. Coexistence of *B holmesii* and *B. parapertussis* was not observed in this study.

We retained no difference in the demographics, clinical features, and vaccination status among patients infected by *B. holmesii* and *B. pertussis*, as reported previously [9].

RT-PCR provides a sensitive and specific diagnosis of *B. pertussis* infections in infected cases for a period not exceeding 3 to 4 weeks maximum, beyond that, it will be negative [20]. For this reason for adolescents or adults coughing more than 3 weeks serology can be used.

In this study, anti-PT IgG concentrations were measured using a commercial kit, comparable to other serological kits tested previously [19]. This kit was reported to have a sensitivity and specificity up to 88 and 100%, respectively [18]. Whereas other techniques, such as agglutination, indirect fluorescence, immunoblotting or complement fixation, are discouraged [30]. If diagnosis cannot be established with certainty, or in case of non- availability of a second serum sample, we used a second commercial kit [19, 20]. This test utilizes purified pertussis toxin as an antigen, allowing quantitative determination of IgA antibodies to Pertussis Toxin according to the first International WHO Standard [31].

Previously, there were opinions that eliminated the measurement of IgA in the serological diagnosis of pertussis [32, 33]. However, a European collaboration [20], suggested that IgA antibodies have a marginal value for the serological diagnosis of pertussis and can be used as an additional method only for testing serum with anti-PT IgG concentrations in ranges that were undetermined which facilitate results interpretation.

Anti-PT IgG / IgA antibodies was confirmed in 55 (39%) cases indicating for an acute infection or recent contact. *B. pertussis* was confirmed by both biological tests (PCR and anti-PT antibodies) in 41 (29%) household members. Twelve (9%) cases were confirmed only by serological tests. These results showed the contribution of serological test to identify some cases that can be misinterpreted as false negatives, especially in people who had delayed their diagnosis or had prolonged cough, those data are similar to those reported by previous studies [18].

A total of 16% (22/140) of the household contacts had anti-PT IgG levels superior than or equal to 40 IU / mL, including 5 cases with anti-PT IgG antibodies superior than or equal to 100 IU / mL and interpreted as an indication of recent *B. pertussis* infection, usually seen in cases diagnosed at the onset of cough, or recently vaccinated. These results are consistent with the previous kinetics of antibody titers after infection: kinetics differ according to whether patients never was in contact with the bacteria before infection or vaccinated or previously infected [34]. 77% (17/22) cases had intermediate levels of anti-PT IgG (> 40 and < 100 IU / mL), indicating possible infection.

Among these 17 suspected IgG tests, 70% (12/17) had IgA titers indicating a recent infection. The other 5 cases had an IgA value of strictly less than 12 IU / ml, and interpreted as the absence of recent infection or past infection or past immunization. Then, when its possible the addition of IgA measurement might be useful.

In our results we detected presence of *B. holmesii* and *B. parapertussis* DNA in NPs from adults. Serology cannot identify these species. Numerous studies have shown that other *Bordetella species* can be the source of infection / carriage in adults. This leads to propose the development of other serological kits intended for the detection of emergent species of *Bordetella*.

Our results showed that *B. pertussis* was detected in 77 (55%) symptomatic household members, 53 (38%) from mothers, 8 (6%) from siblings, 3 (2%) from grandparents and only one from father and in 47 (64%) hospitalized infants and their mothers. This result suggests that mothers were largely the source of the infection and transmitted the disease to their children, this finding confirms other reports [35]. However, in our study, mothers who brought the child to the hospital, were the contaminator in the majority of cases (86%). These mothers have been systematically sampled, when their children were declared as having whooping cough. This could distorts statistical comparisons between household members. Furthermore, we focused mainly on symptomatic case while pertussis case contact could be asymptomatic. Another limitation in the study was about the serological analysis. Indeed, it’s possible to have a high rates of false positives when the diagnosis is basis on serology. When pertussis diagnosis is based on positive serology, there are need to know that the persons were not immunized in the last year because elevated IgG can be result of their vaccination. These information were not explore in our study. We found that in 19 confirmed infants, mothers were not infected by *B. pertussis*, indicating that mothers were not the only possible source of contamination. The contaminator could have been another household members as father, sibling or grandparents [5] [36] [37] [38]. In the US, where whole-cell vaccine is used, a study indicate that the source of infant pertussis has shifted from the mother to the adolescent siblings [39]. Continued monitoring of the source of infant pertussis through surveillance is important, especially as the epidemiology of pertussis changes over the time.

## Conclusion

In conclusion, the results of this study suggest that, despite a high vaccination coverage rate of 95% in primary vaccination in Casablanca, pertussis is not controlled and dangerously present in household contacts of infants. A rapid diagnosis of pertussis in infants using RT-PCR is of high importance in order to treat with macrolides all persons around the infant in order to stop transmission of the disease. Unveiling household contacts pertussis contaminated by RT-PCR alone is less sensitive, especially in who coughed for a long time, it must be completed if it’s negative by serologic diagnosis. This study is the basis of a perennial surveillance in Morocco, not only in Casablanca but in the whole country. Increasing awareness of pertussis among General Practitioners, health care workers but also the Public is also a major objective in our country.

## Data Availability

All the data supporting the conclusions of the present study are included within the manuscript. Supplementary datasets used and/or analyzed during the current study are available from the corresponding author on reasonable request.

## Abbreviations

ELISA: Enzyme linked immuosorbent assay
IR-UHC: Ibn Rochd University Hospital Centre of Casablanca
NPA: Nasopharyngeal aspiration
ptx: pertussis toxin gene
RT-PCR: real time polymerase chain reaction
WHO: World Health Organization
